# Massively parallel CRISPR-assisted homologous recombination enables saturation editing of full-length endogenous genes in yeast

**DOI:** 10.1126/sciadv.adj9382

**Published:** 2024-05-15

**Authors:** Lei Deng, Yi-Lian Zhou, Zhenkun Cai, Jie Zhu, Zenan Li, Zehua Bao

**Affiliations:** ^1^ Key Laboratory of Biomass Chemical Engineering of Ministry of Education, College of Chemical and Biological Engineering, Zhejiang University, Hangzhou 310058, Zhejiang, China.; ^2^ ZJU-Hangzhou Global Scientific and Technological Innovation Center, Zhejiang University, Hangzhou 311215, Zhejiang, China.; ^3^ Institute of Bioengineering, College of Chemical and Biological Engineering, Zhejiang University, Hangzhou 310058, Zhejiang, China.; ^4^ Bota Biosciences, Hangzhou 311222, Zhejiang, China.

## Abstract

Performing saturation editing of chromosomal genes will enable the study of genetic variants in situ and facilitate protein and cell engineering. However, current in vivo editing of endogenous genes either lacks flexibility or is limited to discrete codons and short gene fragments, preventing a comprehensive exploration of genotype-phenotype relationships. To enable facile saturation editing of full-length genes, we used a protospacer adjacent motif–relaxed Cas9 variant and homology-directed repair to achieve above 60% user-defined codon replacement efficiencies in *Saccharomyces cerevisiae* genome. Coupled with massively parallel DNA design and synthesis, we developed a saturation gene editing method termed CRISPR-Cas9– and homology-directed repair–assisted saturation editing (CHASE) and achieved highly saturated codon swapping of long genomic regions. By applying CHASE to massively edit a well-studied global transcription factor gene, we found known and unreported genetic variants affecting an industrially relevant microbial trait. The user-defined codon editing capability and wide targeting windows of CHASE substantially expand the scope of saturation gene editing.

## Teaser

Facile saturation editing of full gene coding sequence with user-defined codon changes was achieved in *Saccharomyces cerevisiae*.

## INTRODUCTION

Understanding the phenotypic impacts of genetic variants is a central goal of molecular genetics and a prerequisite for reliably engineering the field of biology (*1*, *2*). Creating and assaying a large quantity of natural or de novo genetic variants are required for deriving novel understandings and discovering improved enzymes and microbial cell factories (*3*). In vitro saturation mutagenesis is such a technology that has been tremendously successful in mapping protein fitness landscapes and engineering novel protein functions (*4*). However, such derived gene variants are expressed out of their native expression context, either on plasmids or at an ectopic chromosomal locus. This may confound phenotypes by plasmid copy number variations or chromosomal neighborhood effects. To evaluate genetic variants in their native expression context, the “delitto perfetto” technique (*5*) used a two-step selection strategy to knockin site-directed mutations in yeast. A series of selection marker-integrated strains were used to perform saturation mutagenesis, with each strain covering a 200-nucleotide region. Another technique, CRISPR-flippase recognition target (CRISPR-FRT) (*6*), successfully introduced a library of 68 genetic variants along a gene by cleverly targeting two copies of a common sequence flanking a selection marker, which were preinstalled in the *Escherichia coli* knockout collection. These techniques, however, rely on marker-integrated strains and are thus better applied to laboratory strain backgrounds and nonessential gene targets.

To circumvent the use of selection marker-integrated strains, a variety of saturation gene editing (SGE) methods were developed based on genome editing agents, such as Cas9 coupled with homology-directed repair (HDR) (*7*–*13*), EvolvR (Cas9 nickase–DNA polymerase fusions) (*14*), base editors (*15*–*17*), and prime editors (*18*, *19*). Error-prone DNA polymerases introduce random mutations, and base editors introduce limited base conversion types, such as C to T or A to G. Moreover, concurrent conversion of consecutive bases occurs less frequently. HDR and prime editing achieved templated editing of endogenous genomic regions yet with limited targeting windows around the Cas9 targeting loci, which require the presence of a canonical protospacer adjacent motif (PAM) in the form of 5′-NGG.

Recently, several Cas9 variants were engineered to recognize noncanonical and relaxed PAM sequences. Among the most PAM-relaxed Cas9 variants, xCas9-3.7 (*20*), SpCas9-NG (*21*), and SpG (*22*) recognize an NGN PAM, while SpRY (*22*) recognize NRN PAMs and NYN PAMs to a lesser extent. It was shown that xCas9-3.7 exhibited a biased preference for the canonical NGG PAM (*22*), and SpCas9-NG might cleave the guide RNA (gRNA) expression cassette (*23*). While SpG and SpRY exhibited more uniform editing at relaxed PAM sites, no study has evaluated these variants in high-throughput genome editing. To achieve saturation editing of full-length endogenous genes, we sought to assess the capabilities of PAM-relaxed Cas9 variants in HDR-based high-throughput gene editing in *Saccharomyces cerevisiae*, an important model organism for both basic research and applied biotechnology (*24*, *25*).

In this study, we introduce CRISPR-Cas9– and HDR-assisted saturation editing (CHASE) as a novel SGE method. We show that a homologous recombination donor–coupled CRISPR system using an SpG variant exhibited high codon replacement efficiencies for scalable gene editing. When targeted to an endogenous gene, CHASE efficiently generates user-defined codon replacements across gene length with improved coverage. We showcase the utility of CHASE in engineering hyperosmotic stress tolerance, an industrially relevant microbial trait, discovering known and previously unidentified genetic variants in an essential gene with improved fitness under stressed conditions. CHASE is adaptable to both laboratory and industrial genetic backgrounds for massively creating and studying de novo genetic variants in their native expression context, which should hold great promise in strain and protein engineering.

## RESULTS

### Expanding the design space of SGE with CHASE

**Fig. 1.**
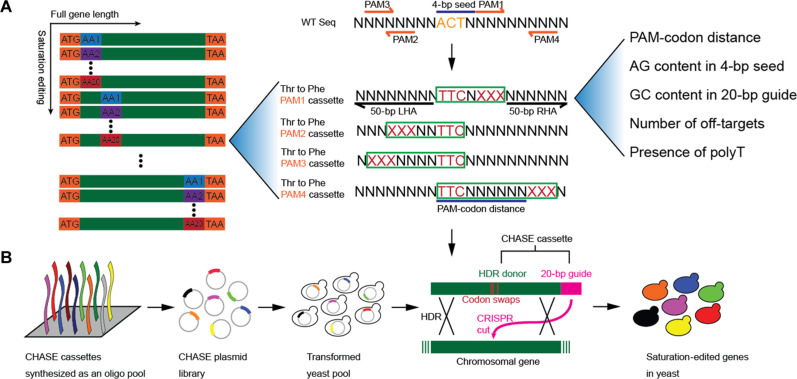
Schematic of CHASE. (**A**) Design of a CHASE library for saturation editing of each AA encoding codon within an ORF. The left panel describes saturation editing of a full-length ORF. Each color block represents a codon encoding a corresponding AA. Each codon is replaced by codons encoding all 20 natural AAs. The middle panel details the CHASE cassette design for mutating a Thr codon ACT to a Phe codon TTC, as an example. Four PAMs were selected from either strand of wild-type DNA. For each PAM, a donor was designed to mutate both the codon and the PAM (green box). Homology arms (50 bp) were partially shown. WT Seq, wild type sequence; XXX, synonymous mutations to eliminate the PAM. LHA, left homology arm. RHA, right homology arm. The right panel describes parameters considered in designing the cassettes. (**B**) The experimental workflow for massively parallel CHASE cassette synthesis, assembly, transformation, and editing. During editing, the 20-bp guide directs a CRISPR cut near the targeted codon and facilitates HDR to enable codon swapping.

We aim to design a CHASE library with editing cassettes replacing each codon in an open reading frame (ORF) to codons encoding all 20 natural amino acids (AAs) (Fig. 1A). For each codon swap, an HDR donor and a corresponding CRISPR guide need to be designed. To do this, we searched for PAMs in close proximity to the target codon. For each candidate PAM, we generated an HDR donor with both mutations to edit the codon and synonymous mutations to eliminate the PAM to prevent Cas9 cutting of the donor and the edited sequence. The donor was fused to the 5′ end of the CRISPR guide sequence to derive the CHASE editing cassette (Fig. 1B), establishing a donor-guide linkage. To ensure efficient editing at a library scale, previously identified parameters were considered for ranking and selecting high-efficiency cassettes (*7*), including PAM-codon distance, purine content in the 4–base pair (bp) seed sequence located 5′ of PAM, GC content in the 20-bp guide sequence, the number of off-targets found in the yeast genome, and the presence of polyT sequences. Cassettes with a short PAM-codon distance, high purine content in 4-bp seed, 30 to 80% GC content in 20-bp guide, and no identical 12-bp seed off-targets were top-ranked. In addition, any cassette with a polyT (a string of five or more T’s) was discarded as it terminates transcription from a type III promoter. For each codon swap, four such top-ranked cassettes were included in the library (Fig. 1A). The CHASE cassette library was massively synthesized, assembled, and transformed into yeast cells to perform SGE (Fig. 1B).

**Fig. 2.**
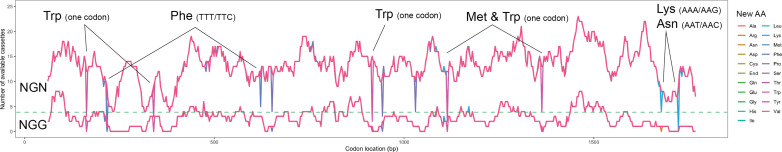
The design landscapes of saturation editing cassettes across the *ADE2* coding region targeting either NGN or NGG PAMs. Numbers of available cassettes for mutating each codon to another new codon are shown. The green dashed line indicates a number of four cassettes. New codons with a substantially lower number of available cassettes in the NGN library were annotated.

We previously demonstrated saturation editing of short (87 bp) genomic regions within an endogenous gene using wild-type Cas9 from *Streptococcus pyogenes* (SpCas9) and HDR (*7*). However, its editing efficiency and editing window were both limited by the requirement for an NGG PAM. Using a Cas9 variant with a relaxed PAM requirement should alleviate these limitations. To assess the degree of editing saturation by such Cas9 variants, we computationally generated two CHASE cassette libraries targeting NGG PAM and the relaxed NGN PAM across the full-length endogenous *ADE2* ORF (Fig. 2). Indeed, for most of the codons, we were only able to design less than four cassettes per codon when NGG PAMs were required. A total of 9.7% of the codons were not editable, and 18.8% of the codons were targeted by only one cassette. Given the chance that any cassette could be inefficient or poorly represented in a library, codons with fewer targeting cassettes are less likely to be edited. In contrast, the library designed with NGN PAMs is greatly expanded. At each codon, more than four cassettes were available for conversion to another codon. Exceptions are Met, Trp, Phe, Lys, and Asn encoding codons, which have fewer cassettes in certain sequence contexts, mainly because only one codon is available for that AA or codon sequences are AT-rich, generating polyT sequences in certain sequence contexts. This in silico analysis showed that a Cas9 variant with a relaxed PAM substantially expands the design space of saturation editing libraries.

### Testing gene knockout efficiencies of SpCas9 variants

To realize CHASE, we first evaluated the gene knockout efficiencies of two PAM-relaxed Cas9 variants, SpG and SpRY, together with a previously identified high-efficiency Cas9 variant SpiCas9 (*26*) (fig. S1). These two variants were selected based on their superior editing efficiencies and uniformity at NGN and NRN/NYN PAMs over other PAM-relaxed Cas9 variants (*22*). We selected a high-efficiency target site (ADE2.a) and a low-efficiency target site (ADE2.b) (*26*) within the endogenous *ADE2* gene to compare the performance of the three variants (fig. S2). HDR donors were designed to introduce a frameshift mutation to the *ADE2* ORF. At both sites, SpG and SpRY exhibited lower editing efficiencies than SpiCas9 (fig. S3). To further improve the editing efficiencies of PAM-relaxed variants, we introduced the mutations of SpiCas9 to SpG and SpRY to derive SpiG and SpiRY, respectively (fig. S1). The mutations of SpiCas9 improved the editing efficiencies of both SpG and SpRY at the two target sites. Notably, the editing efficiencies of SpiG at ADE2.b were close to those of SpiCas9. We further tested editing efficiencies at two noncanonical PAM sites: ADE2.c with a CGA PAM and ADE2.d with a GGT PAM (fig. S2). All five variants edited at ADE2.d, but only SpiG showed minimal editing at ADE2.c, possibly due to the inefficiency of the ADE2.c guide sequence, which is hard to predict when selecting a particular guide sequence (*7*, *26*) (fig. S3). Surprisingly, SpiCas9 was also able to edit at the noncanonical GGT PAM, which echoes a previous study (*27*). At all three editable sites, SpiG exhibited the highest editing efficiencies. We then proceeded to apply SpiG for codon replacements.

### Efficient codon replacement using an NGN-targeting CRISPR system

**Fig. 3.**
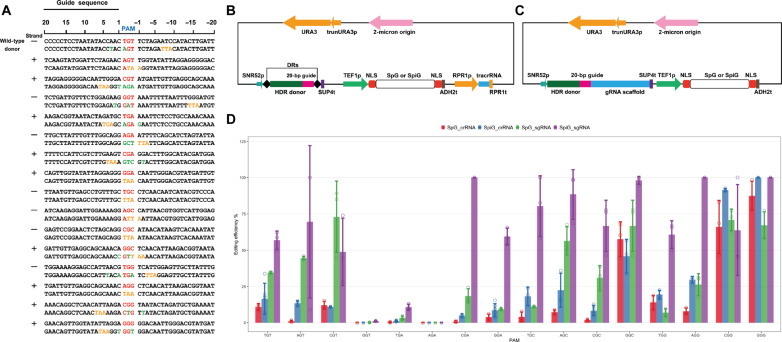
An optimized CRISPR-Cas9 system for precise codon replacement. (**A**) Wild-type *ADE2* sequences and designed donors targeting 16 NGN sites. Red, PAM sequences. Yellow, stop codon mutations. Green, synonymous mutations to mutate the PAM or the guide sequence. Strand from which the guide sequence was selected is denoted on the left. All sequences are oriented in a way that guide sequences from 5′ to 3′. (**B**) Map of crRNA plasmid with SpG or SpiG. trunURA3p, a truncated URA3 promoter. DR, direct repeat. NLS, nuclear localization signal. (**C**) Map of gRNA plasmid with SpG or SpiG. (**D**) Editing efficiencies of SpG and SpiG in combination with a crRNA or a gRNA at the 16 NGN target sites. *n* ≥ 3 biological replicates. Error bars represent standard deviations.

At the high-efficiency site ADE2.a, SpiG edited *ADE2* with efficiencies below 20%, while SpiCas9 edited with efficiencies above 60% (fig. S3). Since the efficiencies of codon replacement were generally lower than gene knockout (*7*), we decided to assess and improve editing efficiencies directly for codon replacement. To fully test the editing capability of the CRISPR-SpiG system targeting NGN sites, we semi-randomly chose 16 CHASE cassettes from the designed NGN library (Fig. 2). The 16 CRISPR guide sequences were from either DNA strand within the 5′ half of the *ADE2* ORF, with each followed by 1 of the 16 NGN PAMs (Fig. 3A, fig. S4, and table S1). For each guide, an HDR donor replaced a nearby codon with a stop codon. In some cases where the stop codon did not eliminate PAM, synonymous mutations were additionally included in the donor to mutate the PAM sequence and/or the protospacer sequence (Fig. 3A). We expressed SpG or SpiG together with each CHASE cassette from a previously developed high–copy number plasmid pCRCT (*26*) (Fig. 3B). This plasmid expressed the CRISPR guide sequence as a CRISPR RNA (crRNA), which was initially designed for multiplex genome editing and showed high editing efficiencies with SpiCas9. For 12 of the 16 cassettes, SpiG exhibited improved editing efficiencies than SpG, consistent with our gene knockout results (Fig. 3D). Among the improved ones, cassettes targeting AGT and CGA were barely editing with SpG but had substantial editing with SpiG. Cassettes targeting CGT and GGC had comparable editing efficiencies. Three of the cassettes (targeting GGT, TGA, and AGA) showed no or low levels of editing with both SpG and SpiG. For the GGT and AGA targeting cassettes, this may be due to a track of T-rich sequence (5′-TTGTTTCT and 5′-TTATTTGTTT, respectively) present in the guide and donor sequences, which might cause premature RNA transcription termination (Fig. 3A). For the TGA targeting cassette, the reasons were not obvious. Despite the substantial improvements, the overall editing efficiencies were below 25% (except for cassettes targeting GGC, CGG, and GGG, which already showed above 60% editing efficiencies with SpG). This indicated that further optimization of the NGN-targeting CRISPR system is needed for high-efficiency saturation editing.

To further improve editing efficiencies, we replaced crRNA and tracrRNA expression cassettes with a single gRNA expression cassette (Fig. 3C) since a single gRNA generally facilitates ribonucleoprotein complex assembly. For SpG, using gRNA increased the editing efficiencies of 12 NGN cassettes as compared to using dual crRNA:tracrRNA (Fig. 3D). For eight cassettes, the effects of gRNA were larger than those of SpiG. Notably, the SpiG-inefficient TGA cassette showed editing when using gRNA, albeit at a low level. For SpiG, using gRNA showed comparable or higher editing efficiencies of all NGN cassettes as compared to the dual RNA approach. When both are coupled with gRNA, SpiG showed higher editing efficiencies than SpG with 12 of the NGN cassettes. The improvements were dramatic with CGA, GGA, TGC, TGG, and AGG cassettes, with an increase from below 25% to 60 to 100%. Again, no combination was able to edit with the GGT or AGA cassette. The editing efficiency of TGA cassette was substantially improved using SpiG and gRNA. In general, codon replacement efficiencies reached above 60% with an appropriate guide sequence. We additionally evaluated the off-target activity of SpiG through whole-genome sequencing of edited colonies by the highly efficient GGG cassette. As compared to the wild-type background, SpiG did not induce substantially more single nucleotide variations (SNVs) or insertions/deletions (InDels) (fig. S5A). Since the 12-bp seed sequence plus NGN PAM of the GGG cassette is unique in the yeast genome, we blasted only the 12-bp seed sequence against the S288C reference genome to find potential off-target sites and compared sites of at least 10-bp matches to the seed with the 103 SpG and SpiG specific SNV/InDels (fig. S5B). Except for the three designed *ADE2* SNPs (Fig. 3A) that mapped to the *ADE2* locus, none of the rest of the 100 SNV/InDels were connected to any potential off-target sites. In summary, an NGN-targeting CRISPR system combining SpiG and gRNA exhibited high codon replacement efficiencies and is suitable for SGE.

### CHASE editing at an endogenous locus is highly saturated

**Fig. 4.**
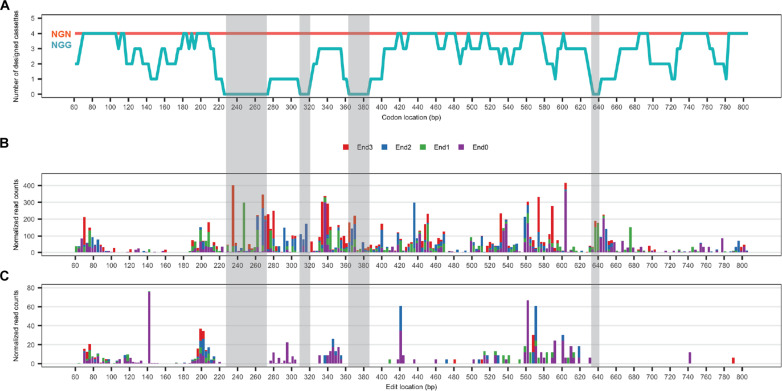
NGS-derived saturation editing results of an *ADE2* genomic region by Cas9 variants. (**A**) The designed NGN and NGG libraries targeting a 5′ 747-bp region of *ADE2*. Orange, number of designed cassettes for each codon in the NGN library. This is a flat line since four cassettes were included for every codon locus. The NGN library includes 996 unique genetic variants. Cyan, number of designed cassettes for each codon in the NGG library. The NGG library includes 618 unique genetic variants. Shaded regions denote codons that are not targeted in the NGG library. (**B**) Editing observed from the NGN library. (**C**) Editing observed from the NGG library. End0, End1, End2, and End3 denote four stop codon swapping edits at each codon location.

We then assessed the saturation editing capability of the optimized CRISPR system at the endogenous *ADE2* locus. For a direct comparison, we synthesized two CHASE libraries aiming to replace each codon encoding the N-terminal 249 AA residues (excluding the start codon) to a stop codon so that editing efficiencies could be monitored by colony color. One library contains CHASE cassettes targeting NGN PAMs and was cloned to the vector expressing SpiG or SpG. For each codon, we included four top-ranked cassettes so that the effects of possible inefficient guides (Fig. 3D and fig. S3) could be compensated by redundant guides. Thus, this NGN library contains 996 CHASE cassettes (table S2) corresponding to 996 genetic variants. The other library contains cassettes targeting NGG PAMs and was cloned to the vector expressing SpiCas9. We were able to design 618 NGG cassettes in total, corresponding to 618 genetic variants, covering 217 (87%) codons (table S3). Among the targetable codons, 181 (73%) codons are targeted by two or more cassettes. Specifically, codons encoding AAs Leu^57^ to Ile^73^, Gln^85^ to His^88^, Ile^103^ to Lys^110^, and Ala^193^ to Met^195^ were not targetable (Fig. 4A). We transformed each library to both a laboratory haploid strain BY4741 and an industrial diploid strain Ethanol Red (ER) (*28*) to assess editing efficiencies at a library scale (fig. S6). Consistent with results obtained from individual editing cassettes (Fig. 3D), SpiG outperformed SpG in both strains with editing efficiencies around 40%. In both strains, editing efficiencies of SpiG were higher than or comparable to those of SpiCas9. We then sequenced the *ADE2* loci of edited colonies from four samples to assess the accuracy of editing (fig. S7). Eight of 10 BY4741 colonies showed perfect editing with mutations matching a designed cassette in the library (fig. S8). In ER, 5 of 10 colonies from the SpiG sample showed perfect editing. One colony had imperfect editing with codon mutations but not PAM mutations. We did not observe mixed genotypes from Sanger sequencing of ER mutants (fig. S7). This indicated that both alleles were edited. We previously demonstrated close to 100% multiplex editing efficiencies in haploid *S. cerevisiae* strains using CRISPR (*26*). We believe that the homogeneous editing observed in diploid ER was due to the high efficiency of the CRISPR system at editing multiple loci. Thus, CHASE also holds potential for applications in other polyploid *S. cerevisiae* strains. Overall, high editing efficiencies and accuracies were achieved with CHASE cassette libraries.

We next analyzed pooled genomic editing at *ADE2* by next-generation sequencing of amplified genomic fragments from edited populations. An edit was counted only when all observed base substitutions matched a designed CHASE cassette in the respective library to distinguish it from sequencing errors. In BY4741, we detected perfect editing at 212 codons by the NGN library, constituting 85.8% of 247 sequenced target codons (two codons encoding Leu^84^ and Met^167^ were not covered by NGS due to primer design; Fig. 4B). This percentage may be higher if more NGS reads were generated and analyzed. Among the edited codons, 65 codons were edited by one CHASE cassette, while 147 codons were edited by two or more cassettes, indicating a high degree of functional cassette redundancy. In ER, 194 codons (78.5%) were edited with 139 of them edited by two or more CHASE cassettes (fig. S9). A total of 180 codons were edited in both BY4741 and ER, and 46 codons were edited in only one strain, which brings the total number of edited codons to 226 (91.5%). In contrast, only 95 codons (38.5%) were edited in BY4741 by the NGG library (Fig. 4C). Among these codons, 57 of them were edited by one cassette, which indicated a relatively low degree of functional cassette redundancy. Notably, we did not detect editing at the four regions with zero designed NGG CHASE cassettes (Leu^57^ to Ile^73^, Gln^85^ to His^88^, Ile^103^ to Lys^110^, and Ala^193^ to Met^195^). At the same regions, saturation editing was achieved with the NGN library (shaded regions in Fig. 4). These results indicate that CHASE editing at an endogenous locus is highly saturated with a several hundred base pair targeting window and improved codon coverage.

### Saturation editing of a global transcription factor gene enables trait improvement

**Fig. 5.**
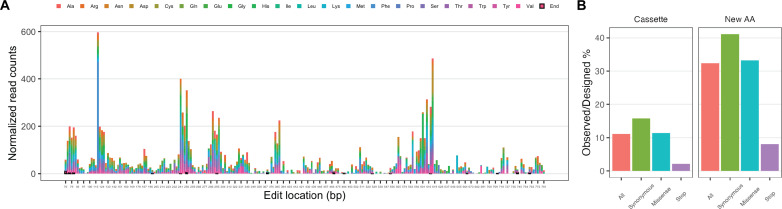
NGS-derived saturation editing results of full-length endogenous *SPT15* gene by CHASE. (**A**) Distribution of target codon edits along the coding sequence. The color scheme denotes swapped new codons encoding each AA. Replaced stop codons are denoted with bold black borders. (**B**) Summary and categorization of *SPT15* NGS data. Left panel, percentage of observed versus designed unique edits corresponding to CHASE cassettes categorized as all mutations, synonymous mutations, missense mutations, and stop mutations; Right panel, percentage of observed versus designed unique new AAs in each category based on the same data from the left panel.

Having demonstrated saturation stop codon swapping at a metabolic gene, we applied CHASE to global transcription machinery engineering (*29*) for tolerance trait improvement by editing the TATA box–binding protein (TBP; 240 AAs) gene *SPT15*. This *SPT15* CHASE library contains 19,967 cassettes, corresponding to 19,967 unique genetic variants, replacing each of 239 non–start codons to codons encoding all 20 natural AAs (including a synonymous design) and a stop codon (table S4). The design success rate was 99.5%, with 109 cassettes missing (table S5) due to similar reasons as in the in silico analysis (Fig. 2). After editing, we sequenced the full-length *SPT15* ORF by NGS and mapped reads against the designed library. We observed diversified editing at 224 target codons of total 239 targeted codons (93.7%) along the full sequence (Fig. 5A). Surprisingly, we observed events of stop codon incorporation at some codon locations of this essential gene (Fig. 5A and fig. S10). Nonetheless, stop codon mutations were less frequently observed as compared to synonymous mutations and missense mutations (Fig. 5B and fig. S11), suggesting their deleterious effects. Possible reasons for survival of stop codon mutants may be the translational readthrough of single stop codons (*30*) or the presence of nonsense suppressor mutations, among others. Two consecutive stop codons may be designed for completely knocking out gene functions in future CHASE experiments. We noted that designing multiple redundant CHASE cassettes encoding each codon swap is necessary to improve the codon coverage of saturation editing as designing one cassette abolished editing at a few codon locations (fig. S12). CHASE was also able to edit wild-type codons encoding each of the 20 AAs (fig. S13). Overall, we observed 11.1% of all possible edits corresponding to individual designed CHASE cassettes, which translates to 32.4% of all possible AA swaps (Fig. 5B). The observed percentage of synonymous AA swapping is 41.1%, which might represent the achieved degree of diversified editing, in this case, regarding that most synonymous mutations are neutral.

**Fig. 6.**
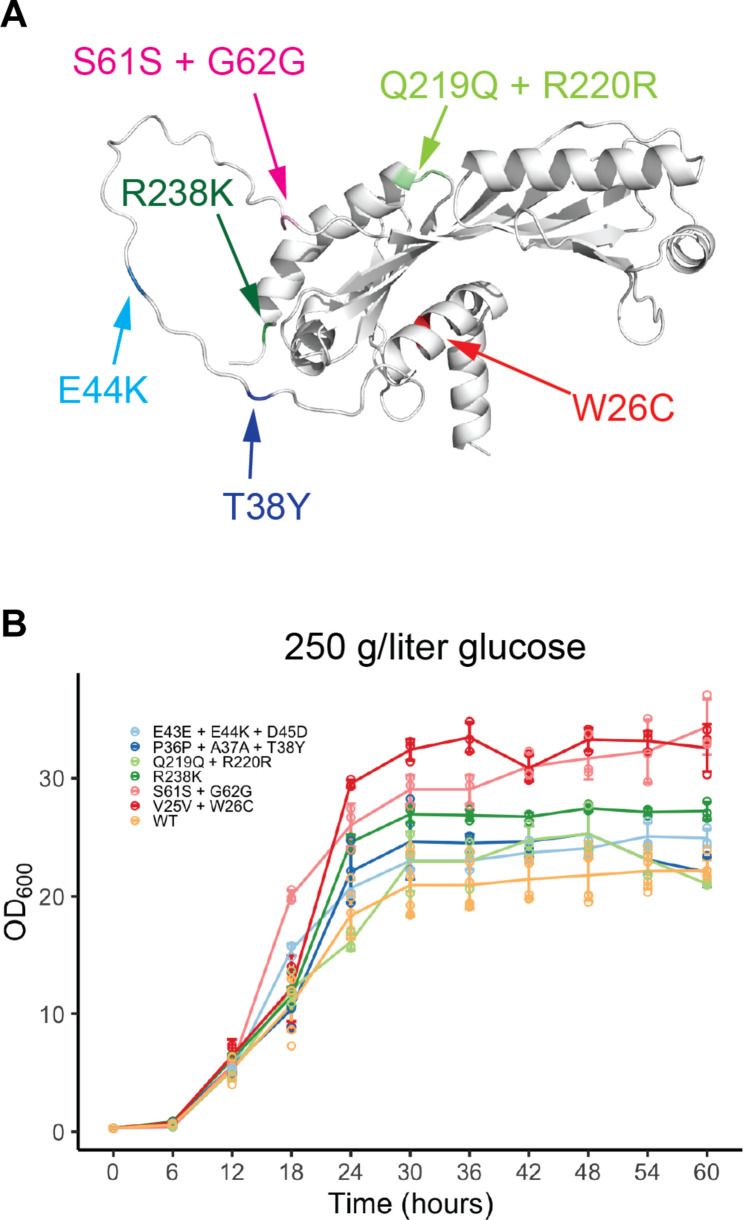
Hyperosmotic stress–tolerant mutants screened from the *SPT15* CHASE library. (**A**) Predicted TBP structure showing the locations of stress-tolerant mutations. (**B**) Growth profiles of reconstructed *SPT15* mutants in YPAD with glucose (250 g/liter). Missense and synonymous mutations for each mutant are shown. WT, wild type. *n* = 3 biological replicates. Error bars represent standard deviations.

We then subjected the CHASE edited yeast cells to growth in liquid culture under glucose (250 g/liter) hyperosmotic stress conditions and subsequently plated cells on stress plates. A total of 150 larger colonies were selected and sequenced to identify survived *SPT15* mutants. Six *SPT15* mutants were identified. Among them, we were able to recover the mutant R238K that was previously identified to confer growth advantage under a high glucose concentration (*31*). In addition, we identified several previously unkown mutants, including W26C, T38Y, E44K, and two synonymous mutants (Fig. 6A and fig. S14). Notably, T38Y and S61S are each due to two and three consecutive nucleotide changes, which is made accessible by saturation mutagenesis and less likely to occur using random mutagenesis techniques. To rule out the possible effects of random adaptive mutations at other genomic loci, we reconstructed these strains by CRISPR editing in a clean background and confirmed that these mutants retained growth advantage over wild-type strains (Fig. 6B) without an adaptation process under stress conditions. We further examined the three top-performing mutants, R238K, W26C, and S61S + G62G, through whole-genome sequencing. We did not observe second-site mutations for R238K and W26C, which indicated direct relationships between these genotypes and fitness phenotypes (table S6 and fig. S15). For S61S + G62G, we observed three second-site mutations not observed in the BY4741 background (table S6). Similar sequences matching the guide sequence were not found at these three loci. Upon recreating S61S + G62G from a parent without these second-site mutations, we again observed substantially increased growth as compared to the wild type (fig. S16). In addition, we recreated S61S + G62G using an alternative gRNA and confirmed its increased growth (fig. S16). Since two gRNAs are not likely to generate the same off-target mutations, we conclude that the growth advantage was due to the *SPT15* mutation. All three previously unidentified missense mutations are in the nonconserved N-terminal domain, suggesting that this domain is involved in certain biological processes related to fitness and warrants further study. The two synonymous mutants also showed improved fitness, especially S61S + G62G, which is comparable to the fittest missense mutant W26C. Although synonymous mutations are commonly regarded as neutral, they might affect multiple gene expression processes including transcription, translation, or even protein folding processes (*32*, *33*) and hence exert downstream effects. For engineering of complex cellular traits such as tolerance, synonymous mutations should thus also be considered as potentially beneficial sequence diversity. This genetic screening experiment validated CHASE in creating de novo mutations for improving an industrially relevant cellular trait.

## DISCUSSION

CHASE is a novel SGE method that substantially expands the editing window of chromosomal genes. The classical delitto perfetto technique and the CRISPR-FRT technique, although generated diversity across a gene, were less flexible in certain application scenarios that require an iterable workflow or industrial strain backgrounds. Other SGE methods typically edit a target window around 100 bp (*8*, *14*, *34*–*36*), limiting the number of candidate AA residues or DNA elements that can be investigated in a single experiment. CHASE was demonstrated to edit endogenous genes of above 700 bp, increasing the length of target window by an order of magnitude. Further increasing the scale of library creation and screening readily allows CHASE to investigate kilo-bp regions.

The seemingly low frequency of found *SPT15* mutations (6 of 150 screened colonies) may be due to several reasons. First, a portion of designed *SPT15* mutations are lethal and thus not present in the edited yeast library since *SPT15* is an essential gene. One indication of evidence is that we observed a lower percentage of missense mutations than synonymous mutations in the edited population (Fig. 5B). Second, a portion of viable *SPT15* mutants may exhibit growth defects as compared to the wild type under a normal growth condition. They will form smaller colonies as compared to the wild type after transformation and plating. Thus, after pooling of the colonies, their representation in the mixed cell population will be low. Third, beneficial mutations under glucose (250 g/liter) stress conditions may be inherently rare. Last, we suspect that the stress level in our experiment was not high enough to inhibit wild-type growth to a level that more beneficial mutations can be found by sampling a reasonable number of colonies. A higher glucose concentration or multiple rounds of enrichment may be applied to increase the selection stringency in future experiments. Nevertheless, the current experiment successfully identified a reported mutant and several previously unidentified mutants, which sufficed the strain optimization purpose of obtaining improved strains if a holistic view of the fitness landscape is not of primary interest. Potential library bias due to different colony sizes may be less of a concern when applying CHASE to nonessential genes or genes whose knockout does not cause a growth defect. Alternatively, sequencing the enriched libraries by NGS will help get a more comprehensive assessment of fitness effects of all possible mutations if this is desired.

Recent advancements in genome editing technologies put an emphasis on non–HDR-based mechanisms, such as base editing (*37*) and prime editing (*38*), which avoid risks of double-strand DNA breaks. Theoretically, gRNAs can be designed to tile a target sequence for SGE. However, most current dual base editing induces only a subset of base conversions in a narrow sequence window (*39*–*42*), which renders swapping of codons with two or three base differences infeasible. Saturation prime editing is capable of any codon replacement by synthesizing customized prime editing templates. However, a current study indicates that efficient prime editing gRNAs are rare within a target sequence despite many efforts on prime editing design optimization, limiting the target window of saturation editing (*18*). CHASE is based on high homologous recombination efficiency of *S. cerevisiae* hosts, alleviating the above limitations in base conversion types and target window length. Since *S. cerevisiae* is a model organism of human biology (*43*–*45*), disease-related human genes can be scanned, and variants can be studied using CHASE.

As a library generation method, CHASE is suitable for directed evolution of enzymes and microbial cell factories. Current in vivo evolution methods rely on error-prone DNA polymerases or RNA polymerases (*46*). Although these methods continuously diversify a full-length gene, they typically suffer from certain base conversion bias and lack the flexibility of user-defined selection of target fragments or AA residues. In addition, except for Cas9-based methods such as EvolvR (*14*), other methods like OrthoRep (*47*) and In vivo Continuous Evolution (ICE) (*48*) require an orthogonal replication or transcription system that is driven by exogenous regulatory parts. As a result, only transgenes but not intrinsically regulated endogenous genes can be evolved. In this study, we applied CHASE to evolve an endogenous global transcription factor in situ in a single round of screening. Since editing templates were user-defined, mutation types and target residues can be customized in future CHASE experiments where rationally selected sites are desired. Compared to traditional global transcription machinery engineering that diversifies an episomal transgene in vitro using error-prone polymerase chain reaction (PCR), CHASE directly installs and assays mutations in a native chromosomal context, which should permit more reliable strain engineering by simultaneously accounting for chromosomal effects and eliminating plasmid copy number variations. By successive transformations of editing cassettes, CHASE omits target gene extraction and in vitro mutagenesis, the most laborious parts of a conventional directed evolution cycle. Coupled with laboratory automation of yeast culturing and transformation (*49*, *50*), we envision iteration of CHASE for high-throughput protein and strain evolution with minimal human intervention.

## MATERIALS AND METHODS

### Strains and media

*E. coli* Trelief5α (catalog no. TSC-C01, Tsingke, Hangzhou, China) and DH5α (catalog no. 9027, Takara Biotechnology, Dalian, China) were used as the cloning hosts for plasmid manipulation and construction and cultivated in LB media [tryptone (10 g/liter), NaCl (10 g/liter), and yeast extract (5 g/liter)] supplemented with ampicillin (100 μg/ml) at 37°C. SOC medium (LB medium supplemented with 2.5 mM KCl, 10 mM MgCl_2_, and 20 mM glucose) was used as the recovery medium for heat shock–transformed *E. coli*. The *S. cerevisiae* strain BY4741 (MATa *his3Δ0 leu2Δ0 met15Δ0 ura3Δ0*) and diploid ER (*his3Δ0 leu2Δ0 trp1Δ0 ura3Δ0*) were used in this study for assessing *ADE2* gene editing efficiency. YPD medium [tryptone (20 g/liter), glucose (20 g/liter), and yeast extract (10 g/liter)] was used to grow yeast cells before transformation. After transformation, cells were grown in synthetic complete minus uracil (SC-U) medium. SC-U agar plates with low (10 mg/liter) adenine hemisulfate were used to screen for *ADE2* edited cells.

### Editing cassette design and synthesis

Cassettes within a library were designed in high throughput by running in-house Python scripts. Available guides and corresponding PAMs across an ORF sequence were first generated using the CRISPRdirect online tool (*51*). For each target codon, PAMs within a 20-bp distance were selected. For each selected PAM, an editing donor was designed to replace the original codon with an intended codon and simultaneously eliminate the PAM by synonymous mutations. The donor contains a left homology arm and a right homology arm of 50 bp in length for maximal recombination efficiencies. If the PAM is selected from the minus strand, then the donor sequence is reverse-complemented to minimize hairpin formation with the guide sequence. The full cassette is assembled by adding the 20-bp guide sequence to the 3′ end of the donor sequence. All designed cassettes for a target codon were then sorted based on PAM-codon distance, AG content in 4-bp seed, GC content in the guide, and the number of potential off-targets. Cassettes with polyT tracks were filtered out since these sequences cause early termination of crRNA/gRNA expression. After sorting and filtering, up to four top-ranking cassettes were included in the final library for synthesis. Flanking sequences for Gibson assembly were added to each cassette. All libraries were commercially synthesized as oligonucleotide pools (Dynegene Technologies, Hangzhou, China).

Individual cassettes were similarly designed to incorporate an 8-bp deletion for *ADE2* knockout or mutations for codon swapping between the two homology arms. The two homology arms were amplified from yeast genomic DNA with primers containing flanking sequences, the edits, and the guide sequence (table S7). The full cassette was assembled by overlapping the PCR of the two homology arm fragments.

### Plasmid construction

All plasmids for yeast genome editing were constructed with pCRCT (Addgene no. 60621) as the initial template. For gRNA receiver plasmid, the tracrRNA expression cassette was deleted by PCR amplification and circularization of the remaining pCRCT backbone. Then, the gRNA scaffold sequence was inserted between the SNR52 promoter and SUP4 terminator by Gibson assembly to replace the direct repeats. DNA fragments containing mutations for SpG, SpiG, SpRY, and SpiRY were yeast codon–optimized, synthesized (Tsingke, Hangzhou, China), and constructed into pCRCT by replacing corresponding iCas9 sequences. For crRNA plasmids, the donor and guide sequence fragment with flanking BsaI restriction sites was assembled with appropriate Cas9 variant receiver plasmids by Golden Gate assembly (*52*). For gRNA plasmids, the donor and guide sequence fragment was assembled with appropriate Cas9 variant receiver plasmids by Gibson assembly (catalog no. E2611, NEB, Hangzhou, China).

### Construction of CHASE plasmid libraries

Each oligonucleotide pool was dissolved into nuclease-free distilled water. The dissolved oligonucleotide pool (1 ng) was amplified using KOD -Plus- Neo polymerase (catalog no. KOD-401, TOYOBO Shanghai, Japan) in a 50-μl total reaction volume with an initial denaturation period of 94°C for 5 min, then 24 cycles of 98°C for 10 s, 51°C for 30 s, and 68°C for 6 s, and finally 68°C for 10 min and held at 4°C. PCR products were cleaned with a PCR purification kit (catalog no. K0702, Thermo Fisher Scientific, Hangzhou, China). Purified PCR products (100 ng) were assembled with 190 ng of linearized pCRCT-gRNA scaffold plasmid in a 20-μl reaction volume using Gibson assembly. Fifteen parallel Gibson assembly reactions were performed, and the resultant DNA was purified. Purified products (2.5 μl) were then electroporated into 50-μl DH5α electrocompetent cells (catalog no. 9027, Takara Biotechnology, Dalian, China) using 0.1-cm electroporation cuvettes (Bio-Rad, Hangzhou, China) with the Bio-Rad Gene Pulser electroporator (1.8 kV, 200 ohms, and 25 μF). Up to 30 parallel transformations were conducted. Transformed cells were recovered in prewarmed SOC medium at 220 rpm and 37°C for 1 hour. After recovery, all cultures were mixed, from which 200 μl was serially diluted and plated onto 90-mm LB plates supplemented with ampicillin to estimate transformation efficiencies. The rest of the culture was plated onto LB plates (24.5 cm by 24.5 cm) with ampicillin. All plates were incubated at 37°C overnight. The total number of colony-forming units was estimated to be 1.2 × 10^6^ (SpiG NGN library for *ADE2*), 4.5 × 10^6^ (SpG NGN library for *ADE2*), 2.2 × 10^6^ (SpiCas9 NGG library for *ADE2*), and 1.95 × 10^8^ (*SPT15* library), which represents a 1205-fold, 456-fold, 356-fold, and 9766-fold coverage of the respective library size. Cells were collected, mixed, and plasmids were extracted using a TIANGEN Plasmid Maxi Kit (catalog no. DP117, TIANGEN, Hangzhou, China).

### Yeast transformation

Single plasmid transformation of BY4741 (1 μg of plasmid per transformation) was carried out using the LiAc/SS carrier DNA/PEG method (*53*). After transformation, cells were transferred to 10 ml of SC-U media in a 100-ml baffled flask and cultivated at 250 rpm and 30°C. Every 48 hours, 100 μl of cell culture was transferred into 10 ml of fresh SC-U media for continued outgrowth. The remaining cell culture was discarded.

For library transformation, 25 μg of plasmid library was used per transformation. Up to 30 parallel transformations were conducted. After transformation, cells were resuspended in liquid SC-U media. Three transformations were randomly selected, diluted, and plated onto SC-U plates for assessing the transformation efficiency. The total number of colony-forming units was estimated to be 5.7 × 10^5^ (SpiG NGN library for BY4741 *ADE2*), 5.0 × 10^5^ (SpG NGN library for BY4741 *ADE2*), 4.7 × 10^5^ (SpiCas9 NGG library for BY4741 *ADE2*), 3.8 × 10^5^ (SpiG NGN library for ER *ADE2*), 3.6 × 10^5^ (SpG NGN library for ER *ADE2*), 2.6 × 10^5^ (SpiCas9 NGG library for ER *ADE2*), and 4.9 × 10^6^ (SpiG NGN library for BY4741 *SPT15*), which represents a 574-fold, 496-fold, 765-fold, 384-fold, 357-fold, 446-fold, and 245-fold coverage of the respective library size. For the *SPT15* mutant library, the rest of the transformation resuspension was pooled and plated onto SC-U plates (24.5 cm by 24.5 cm) and incubated at 30°C for 4 days. Colonies were collected for genomic DNA extraction and amplicon sequencing and stored as glycerol stocks. For *ADE2* mutant libraries, the rest of the transformation resuspension was pooled and plated onto SC-U plates (24.5 cm by 24.5 cm) and incubated at 30°C for 4 days. Colonies were then collected for genomic DNA extraction and amplicon sequencing.

### Calculation of *ADE2* gene editing efficiency

To calculate the efficiencies of editing the *ADE2* gene with different plasmid constructs, 200 μl of 10^4^-fold diluted cell culture was plated onto SC-U plates with adenine hemisulfate (10 mg/liter). Cells were allowed to grow for 3 to 4 days on the plate, and the *ADE2* gene editing efficiencies were determined by calculating the percentage of pink or red colonies.

### Yeast colony genotyping

Genomic DNA extracts from selected yeast colonies were prepared with MightyPrep reagent for DNA (catalog no. 9182, Takara Biotechnology, Hangzhou, China) according to the product manual. Gene fragments were amplified from the extracts using Green Taq Mix (catalog no. P131-03, Vazyme, Nanjing, China) and gene-specific primers. PCR products were sent for Sanger sequencing to determine the genotypes.

### Next-generation sequencing

Genomic DNA were extracted using a yeast genomic DNA extraction kit (catalog no. DP307, TIANGEN, Hangzhou, China) from 2 optical density (OD) of cells. For NGS of the edited *ADE2* gene, the targeted 747-bp region was amplified from genomic DNA as three segments of around 280 bp in length. *SPT15* was amplified as two fragments of 400 and 424 bp. All segments were PCR-amplified using the KOD -Plus- Neo polymerase with segment-specific primers (table S7) and 10 ng of extracted DNA as the template. For *ADE2*, the cycling condition for the first segment is 94°C for 5 min, (98°C for 10 s, 51°C for 30 s, and 68°C for 9 s) × 20 cycles, and 68°C for 10 min and held at 4°C; the cycling condition for the second segment is 94°C for 5 min, (98°C for 10 s, 55°C for 30 s, and 68°C for 9 s) × 20 cycles, and 68°C for 10 min and held at 4°C; the cycling condition for the third segment is 94°C for 5 min, (98°C for 10 s, 53°C for 30 s, and 68°C for 9 s) × 20 cycles, and 68°C for 10 min and held at 4°C. For *SPT15*, the cycling condition for the first segment is 94°C for 5 min, (98°C for 10 s, 50°C for 30 s, and 68°C for 12 s) × 20 cycles, and 68°C for 10 min and held at 4°C; the cycling condition for the second segment is 94°C for 5 min, (98°C for 10 s, 55°C for 30 s, and 68°C for 13 s) × 20 cycles, and 68°C for 5 min and held at 4°C. PCR products were cleaned, and the amount of each purified PCR product was at least 150 ng. Each segment was paired-end sequenced on Illumina Novaseq with a 250-bp read length (Azenta, Suzhou, China).

### NGS data processing and analysis

Paired-end reads were first merged using Illuminapairedend in Galaxy (https://usegalaxy.eu) with default settings. Merged reads were then filtered to contain amplification primers, oriented in the same direction, and sorted into different segments. For each segment, reads of correct length and containing forward and reverse primer sequences at two ends were filtered. Filtered reads were checked at each codon position for designed mutations. All found mutations per read were annotated as the matching CHASE cassette. Each cassette was then counted and normalized according to the total reads of each segment using the following equation: normalized read counts = (raw read counts × 1,000,000)/total read counts.

### Screening of stress-tolerant *SPT15* mutants

The glycerol stocks of pooled yeast *SPT15* mutants were thawed on ice. Cells (3.125 OD) was inoculated into 50 ml of SC-U liquid media with glucose (250 g/liter) in a 500-ml baffled flask. Cells were grown at 30°C and 250 rpm in a shaking incubator, and the optical density was monitored periodically until the plateau phase. The cells were then diluted and plated on SC-U glucose (250 g/liter) stress plates. After 4 days of incubation, large colonies were selected for *SPT15* genotyping and growth profile characterization.

### Characterization of *SPT15* mutant strains

BY4741 wild-type or mutant strains were streaked from glycerol stocks onto fresh YPD solid medium and cultured at 30°C. Three independently edited colonies of each strain were inoculated into 10 ml of fresh YPAD medium [YPD supplemented with adenine hemisulfate (10 mg/liter)] in a 100-mL baffled flask and cultured at 30°C and 250 rpm. After 24 hours, cells were inoculated into a 30-ml fresh YPAD medium with glucose (250 g/liter) in a 250-ml baffled flask, with an initial OD_600_ of 0.3. Cell optical densities at 600 nm was determined every 6 hours using a UV-2350 spectrophotometer (Unico, Shanghai, China).

### Whole-genome sequencing

Whole-genome sequencing of *ADE2* edited cells was performed by Illumina Novaseq (Azenta, Suzhou, China). Sequencing library preparations were constructed following the manufacturer’s protocol. Briefly, for each sample, 200 μg of genomic DNA was randomly fragmented by Covaris to an average size of 300 to 350 bp. The fragments were treated with End Prep Enzyme Mix for end repairing, 5′ phosphorylation, and 3′ adenylation to add adaptors to both ends. Size selection of adaptor-ligated DNA was then performed by DNA cleanup beads. Each sample was then amplified by PCR for eight cycles using P5 and P7 primers, with both primers carrying sequences that can anneal with flowcell to perform bridge PCR and P7 primer carrying a six-base index allowing for multiplexing. The PCR products were cleaned up and validated using an Agilent 2100 Bioanalyzer. The qualified libraries were paired-end sequenced with a 150-bp read length on the Illumina Novaseq System. fastp (v0.23.0) was used to remove sequcences of adaptors, PCR primers, reads with more than 14 N bases, and reads with lower than 40% Q20 bases. Pipeline of Sentieon (v202112.02) was used to map clean data to the S288C reference genome (NCBI RefSeq assembly GCF_000146045.2), remove duplication, and call SNV/InDel. Annotation for SNV/InDel was performed by Annovar (v21 Apr 2018).

Whole-genome sequencing of *SPT15* mutants was performed by Illumina MiSeq (Bota Biosciences, Hangzhou, China). For each mutant, genomic DNA from four colonies were extracted and sample quality checked with Nanodrop and quantified by Qubit. Input DNA (100 ~ 500 ng) was used to begin library preparation using Illumina DNA Prep (catalog no. 20018705). Briefly, genomic DNA was tagmented using bead-linked transposomes and tagmentation buffer 1. Post tagmentation cleanup was performed using tagment stop buffer and tagment wash buffer. Tagmented DNA was then amplified using a limited-cycle PCR program and Enhanced PCR Mix. The PCR step adds Index 1 (i7) adapters, Index 2 (i5) adapters, and sequences required for sequencing cluster generation. Libraries were cleaned up using sample purification beads and resuspension buffer. Libraries were then pooled, diluted, spiked with 5% PhiX, and sequenced for 150 cycles on MiSeq. Reads mapping, variant calling, and variant annotation were performed using breseq (https://github.com/barricklab/breseq) and the S288C reference genome (NCBI RefSeq assembly GCF_000146045.2). Considering deviations between the BY4741 genome and the parental S288C reference genome, we benchmarked all identified mutations with two wild-type BY4741 strains. The mutations that occur in any of the wild-type strains were filtered out. An additional filtering was applied to identify the mutations in common for each *SPT15* mutant group.

### TBP structure prediction and mutant mapping

The full-length TBP structure was predicted using a simplified version of AlphaFold v2.3.2. Identified mutants were mapped onto this predicted structure using PyMOL.

### Statistical analysis

Error bars represent standard deviations. The number of biological replicates is indicated in figure legends when appropriate.
