# Supplementary material for: Massively parallel CRISPR-assisted homologous recombination enables saturation editing of full-length endogenous genes in yeast
Source: Sci Adv. 2024 May 15;10(20):eadj9382. doi: 10.1126/sciadv.adj9382 (PMC11095455; doi:10.1126/sciadv.adj9382)
Supplement: Supplementary file 1 — Figs. S1 to S16 Table S5 Legends for tables S1 to S4, S6 and S7 [file sciadv.adj9382_sm.pdf]

Science Advances
AAAS

# Supplementary Materials for

## Massively parallel CRISPR-assisted homologous recombination enables saturation editing of full-length endogenous genes in yeast

Lei Deng *et al.*

Corresponding author: Zehua Bao, zbao@zju.edu.cn

*Sci. Adv.* **10**, eadj9382 (2024)
DOI: 10.1126/sciadv.adj9382

**The PDF file includes:**

Figs. S1 to S16
Table S5
Legends for tables S1 to S4, S6 and S7

**Other Supplementary Material for this manuscript includes the following:**

Tables S1 to S4, S6 and S7

## SUPPLEMENTARY FIGURES

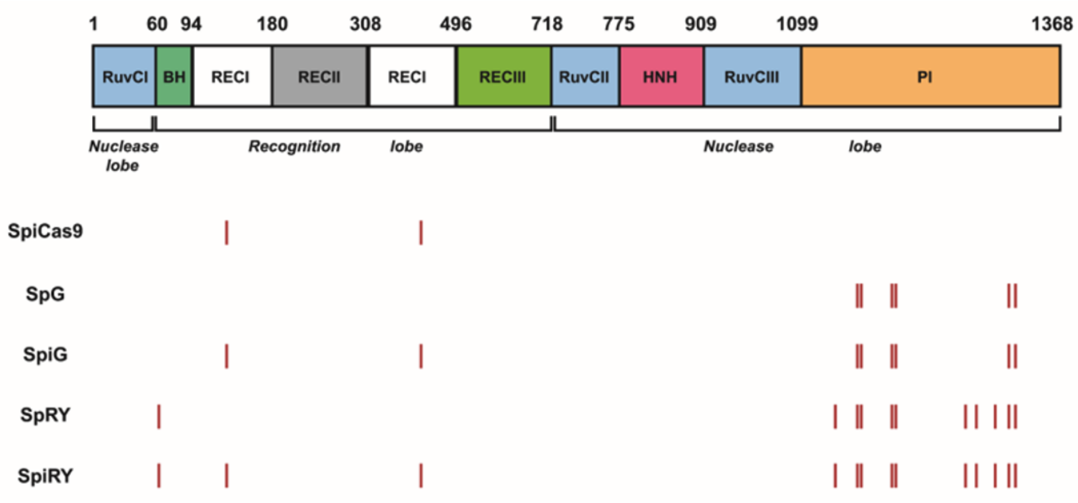


**Figure S1.** Tested Cas9 variants in this study. The domain structure of wild type SpCas9 was shown on the top. Loci of corresponding mutations of each Cas9 variant were marked by red bars at corresponding positions along the Cas9 structure.

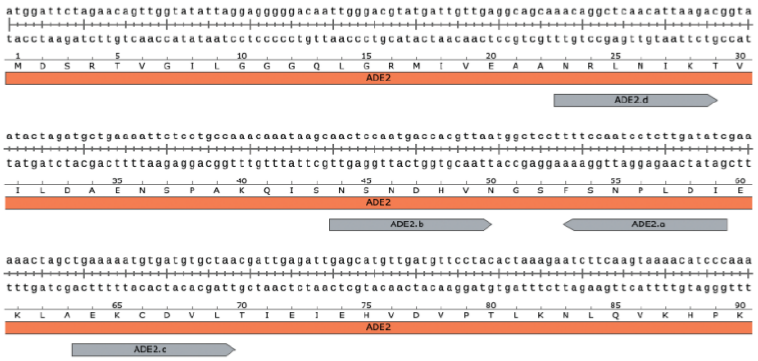


| Target Site | Guide Sequence | PAM |
|---|---|---|
| ADE2.a | GATATCAAGAGGATTGGAAA | AGG |
| ADE2.b | AACTCCAATGACCACGTTAA | TGG |
| ADE2.c | TGAAAAATGTGATGTGCTAA | CGA |
| ADE2.d | AACAGGCTCAACATTAAGAC | GGT |

**Figure S2.** Positions, sequences, and PAMs of 4 *ADE2* targeting guides for gene knockout. In the left panel, part of the *ADE2* 5’ coding sequence was shown. Forward arrows indicate guides from “+” strand, while reverse arrows indicate guides from “-” strand. In the right panel, guide sequences and PAMs were shown in a 5’ to 3’ orientation.

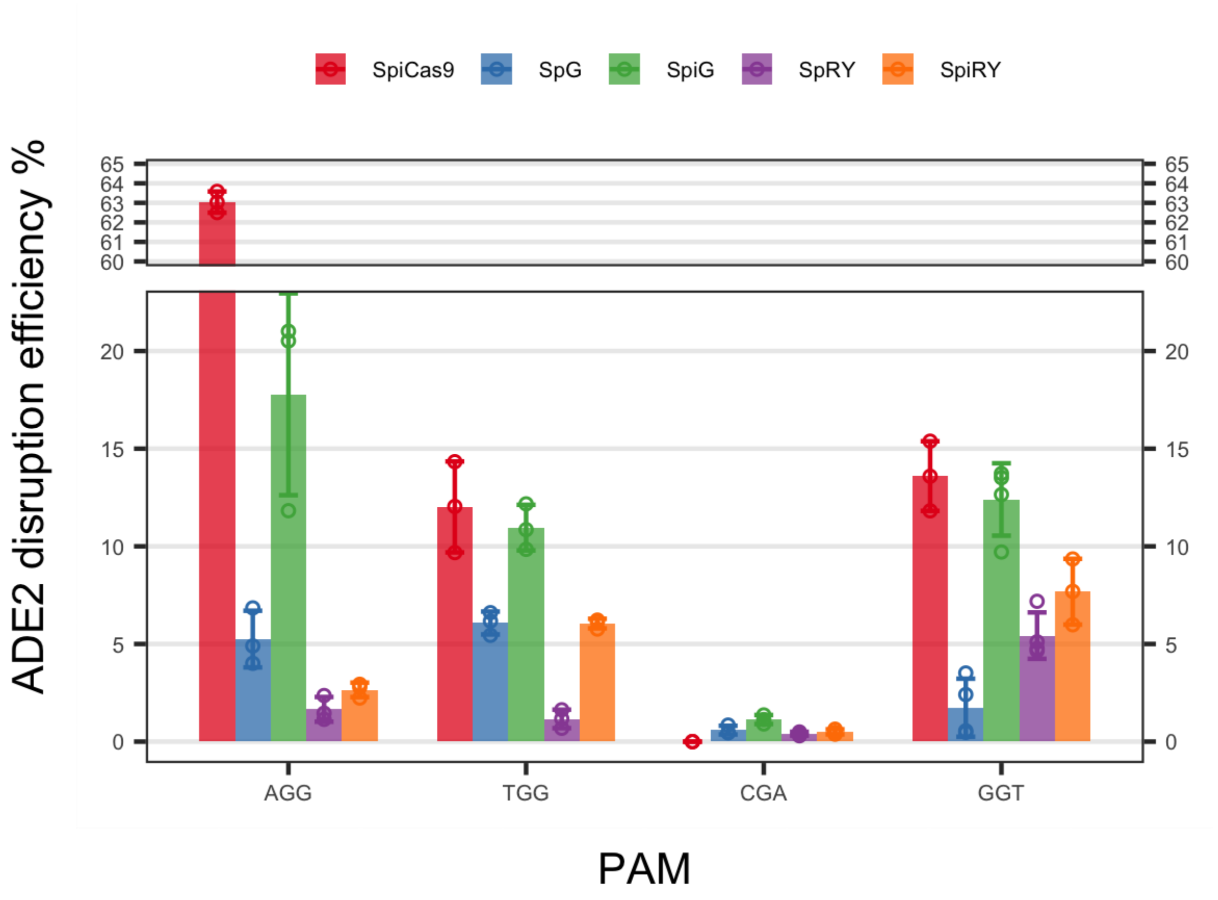


**Figure S3.** *ADE2* gene disruption efficiencies of 5 Cas9 variants at the four target sites with PAM AGG, TGG, CGA, and GGT. n = 3 biological replicates. Error bars represent standard deviations.

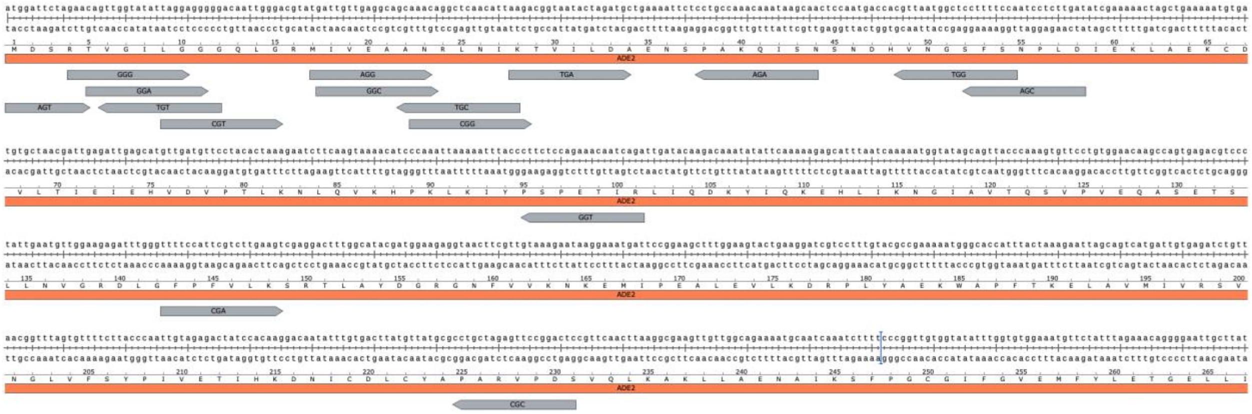

**Figure S4.** Targeting loci of 16 NGN guides used in Figure 3 for stop codon swapping along the *ADE2* ORF (first 800 bp shown). Forward arrows indicate guides from “+” strand, while reverse arrows indicate guides from “-” strand.

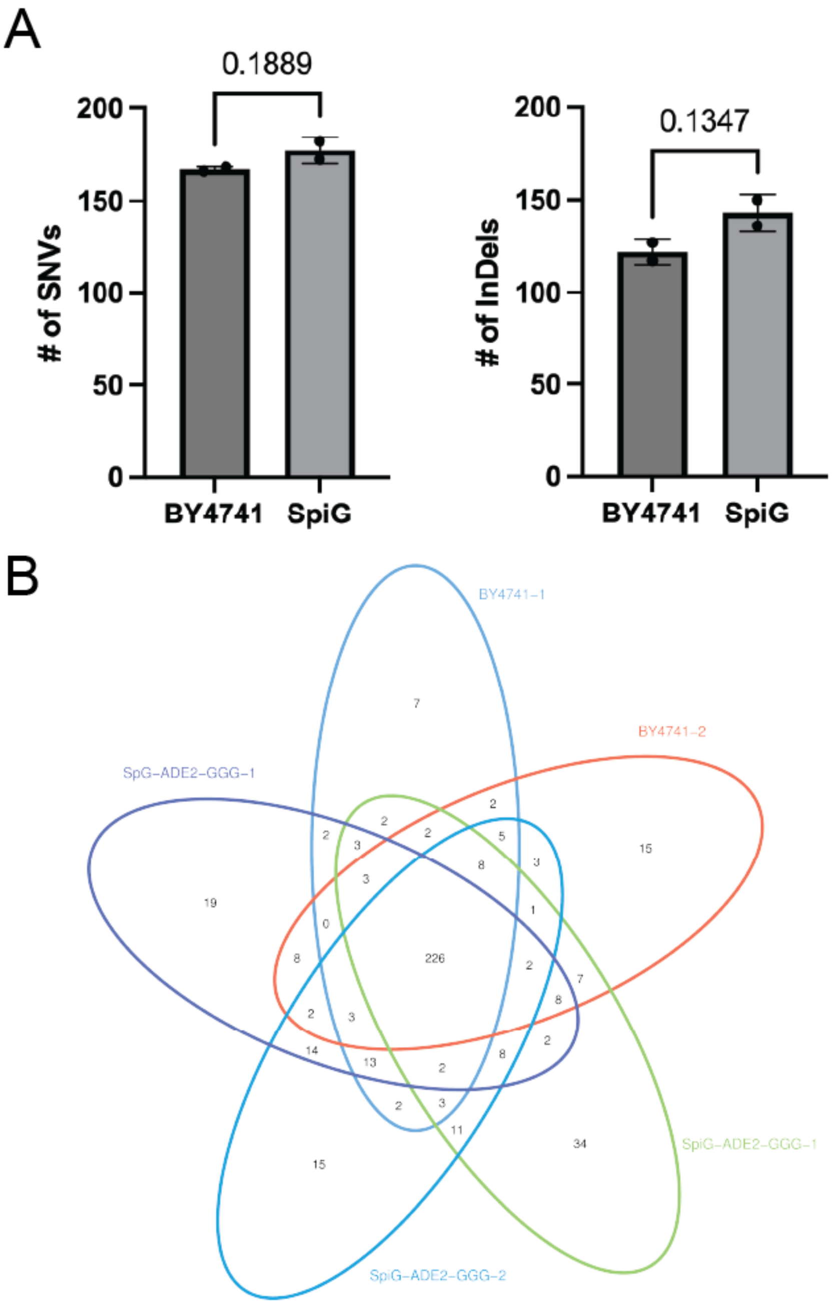


**Figure S5.** Number of SNVs and InDels identified from whole genome sequencing of wild type BY4741 colonies, SpG edited colonies, and SpiG edited colonies. For edited colonies, the GGG CHASE cassette in single gRNA form targeting *ADE2* from figure 3A was used. (A) Comparison of the numbers of identified SNVs and InDels between the BY4741 background and SpiG edited colonies. *P* values from a two-tailed t-test were shown for each comparison. N = 2 biological replicates. Error bars represent standard deviations. (B) A Venn diagram showing the number of differentially identified SNVs/InDels between each group.

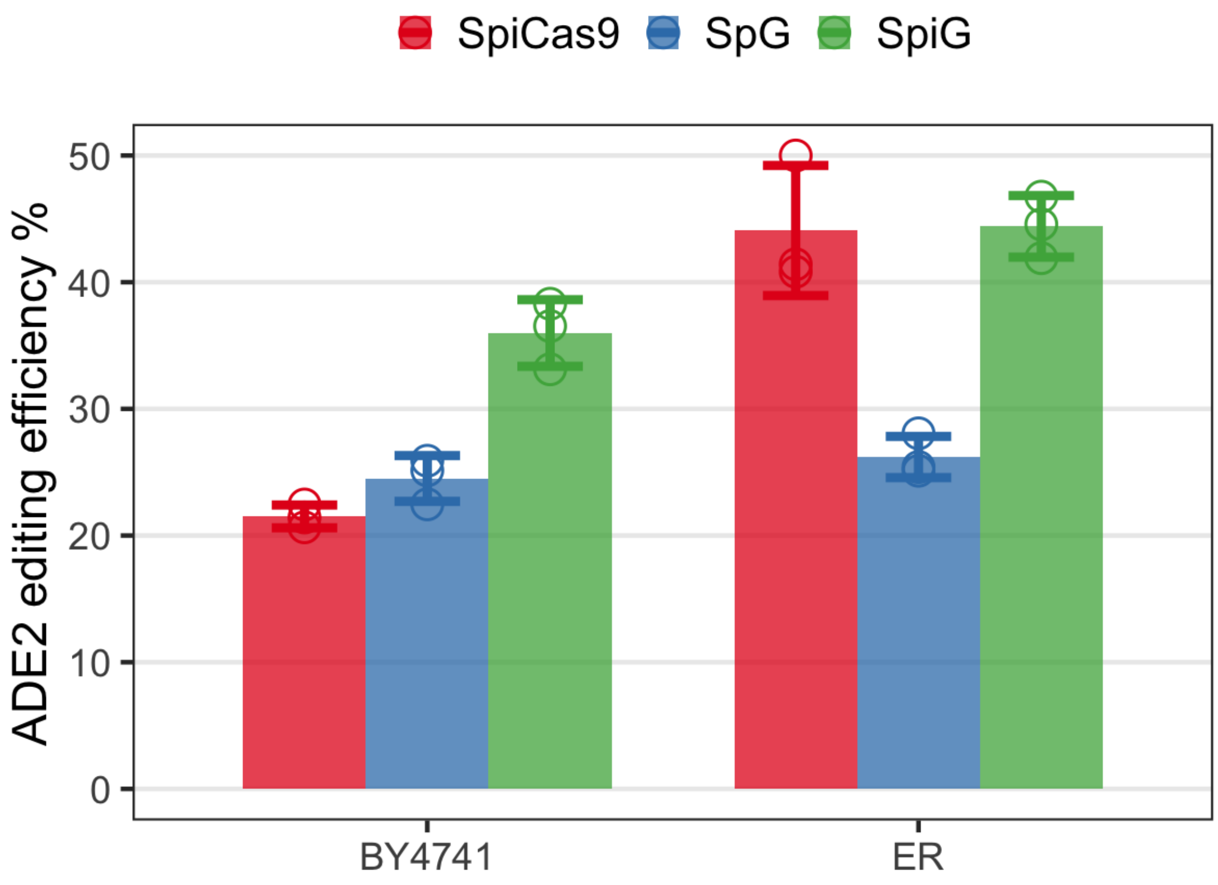


**Figure S6.** *ADE2* gene editing efficiencies of three plasmid libraries after transformation in BY4741 and ER strains. SpiCas9, NGG cassette plasmid library expressing the SpiCas9 protein; SpG and SpiG, NGN cassette plasmid libraries expressing the SpG and SpiG protein, respectively. n = 3 biological replicates. Error bars represent standard deviations.

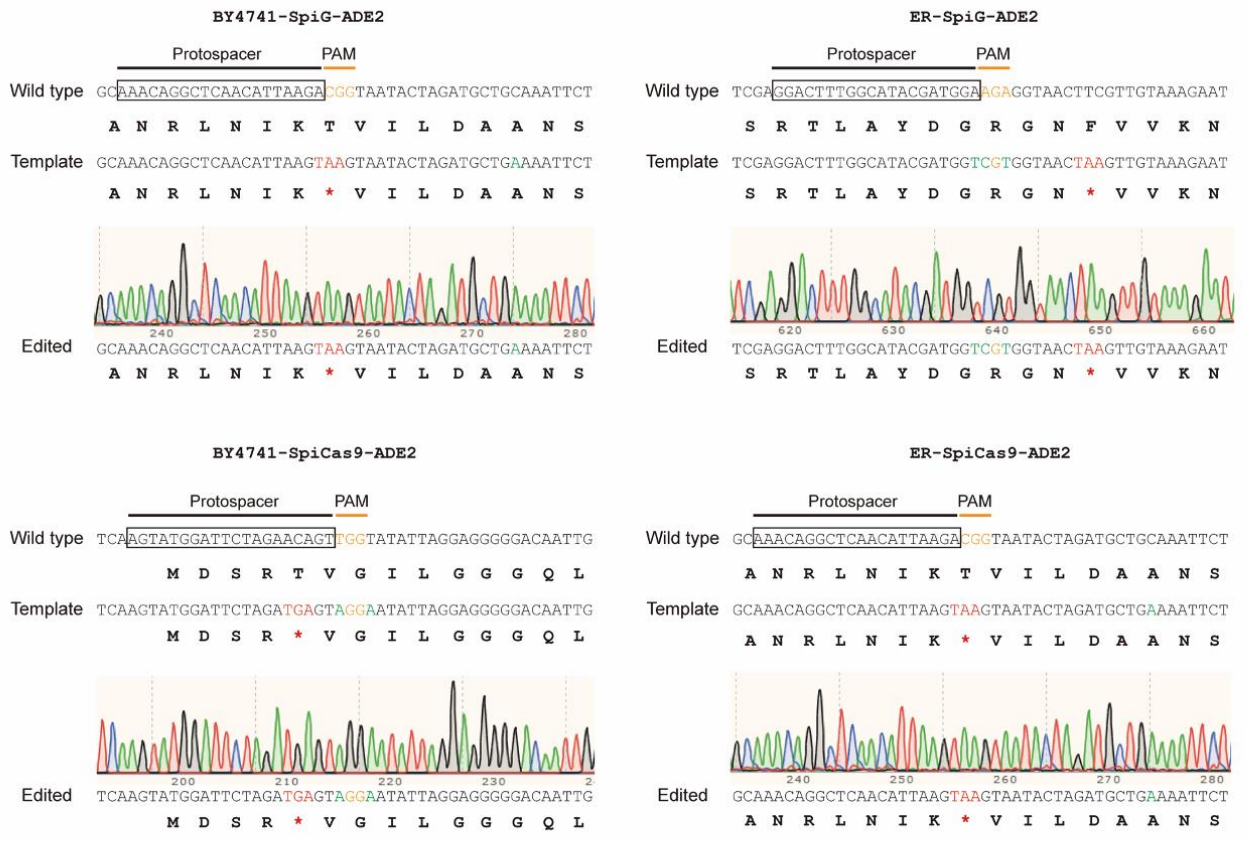


**Figure S7.** Representative Sanger sequencing results of individual *ADE2* edited colonies selected after library transformation. Parent strains and the library transformed were noted above each sequencing result. SpiCas9, NGG cassette plasmid library expressing the SpiCas9 protein; SpiG, NGN cassette plasmid library expressing the SpiG protein. The protospacer and PAM sequences, donor template sequence, and Sanger sequencing trace file were shown for each edited colony. Red fonts indicate stop codon mutations. Green letters indicate designed synonymous mutations. Translated protein sequences were shown under each nucleotide sequence.

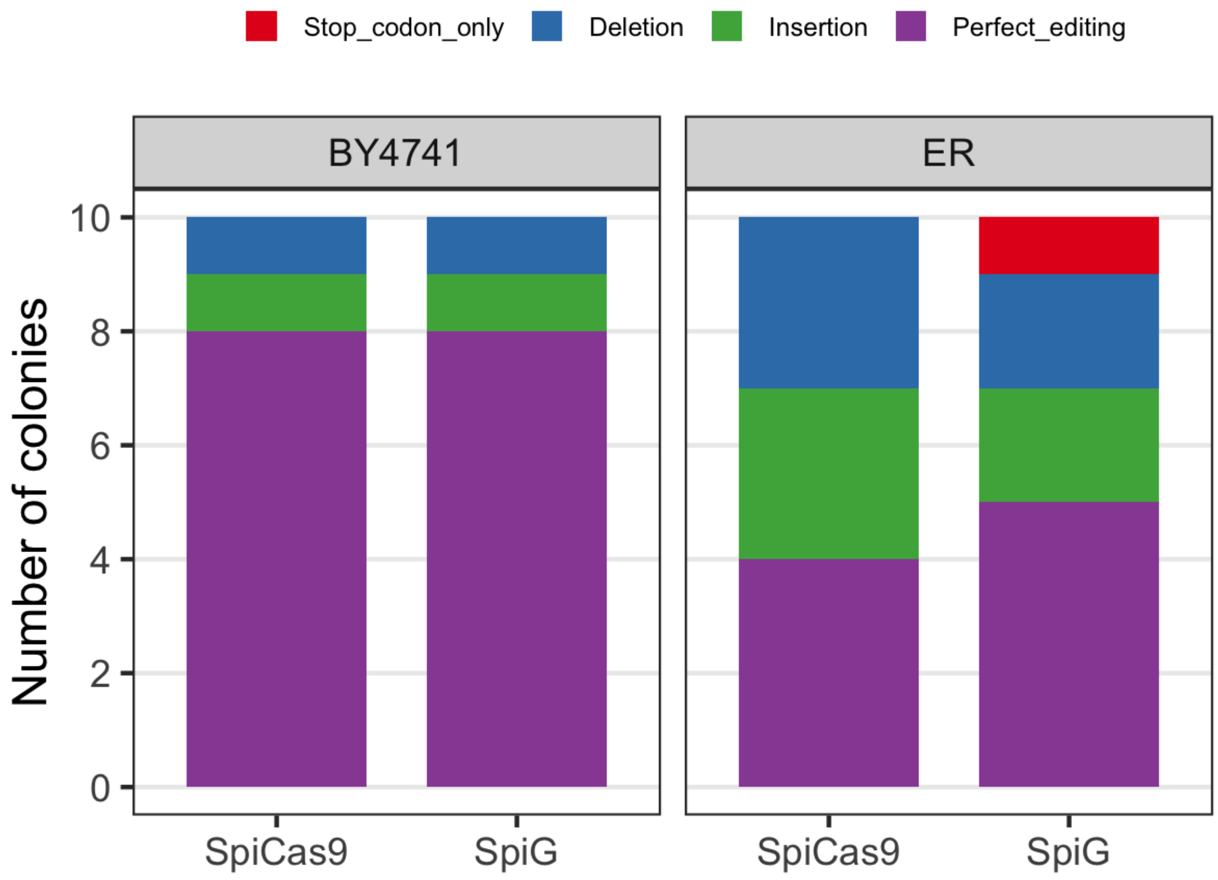


**Figure S8.** Editing accuracies of SpiCas9 and SpiG in BY4741 and ER. 10 pink/red colonies randomly picked from library transformed populations were Sanger sequenced for each group. SpiCas9, NGG cassette plasmid library expressing the SpiCas9 protein; SpiG, NGN cassette plasmid library expressing the SpiG protein. Stop_codon_only, only stop codon mutations but not PAM synonymous mutations were present. Perfect_editing, both stop codon mutations and PAM synonymous mutations were present.

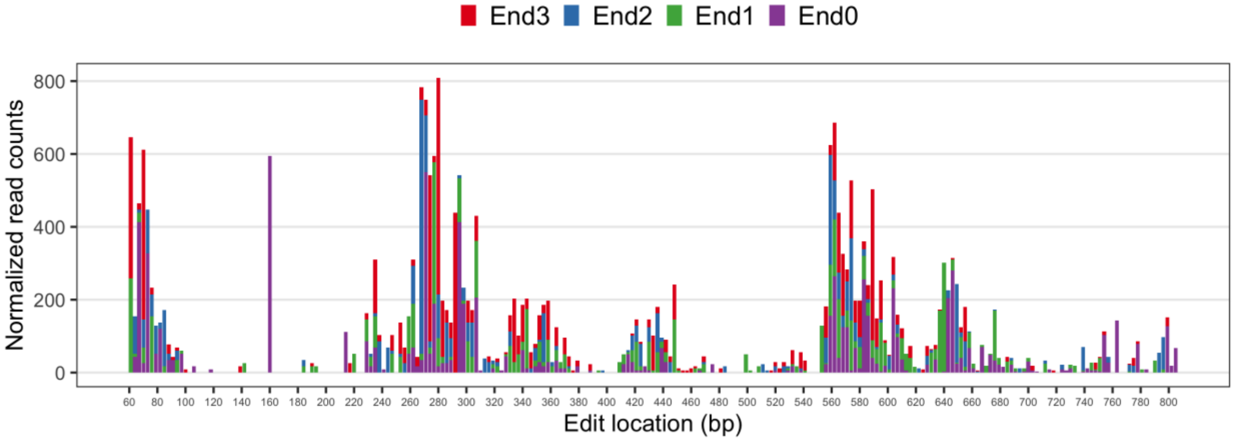


**Figure S9.** Editing of *ADE2* in ER by the NGN library expressing SpiG, analyzed from NGS of the *ADE2* locus. End0, End1, End2, and End3 denote that the wild type codon at each location was edited by the four corresponding CHASE cassettes targeting that location. Their relative abundances were represented by the bar height.

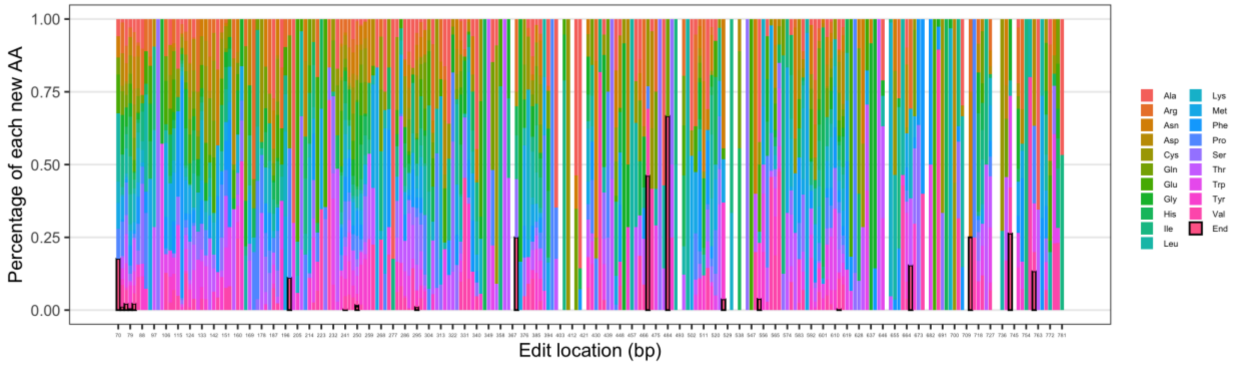


**Figure S10.** Saturation editing of full-length endogenous *SPT15* gene by CHASE as analyzed from NGS data. The y axis denotes relative percentage of swapped new codons encoding each new amino acid at each codon location on the x axis. The color scheme denotes swapped new codons encoding each new amino acid. Observed stop codons are denoted with additional bold black borders.

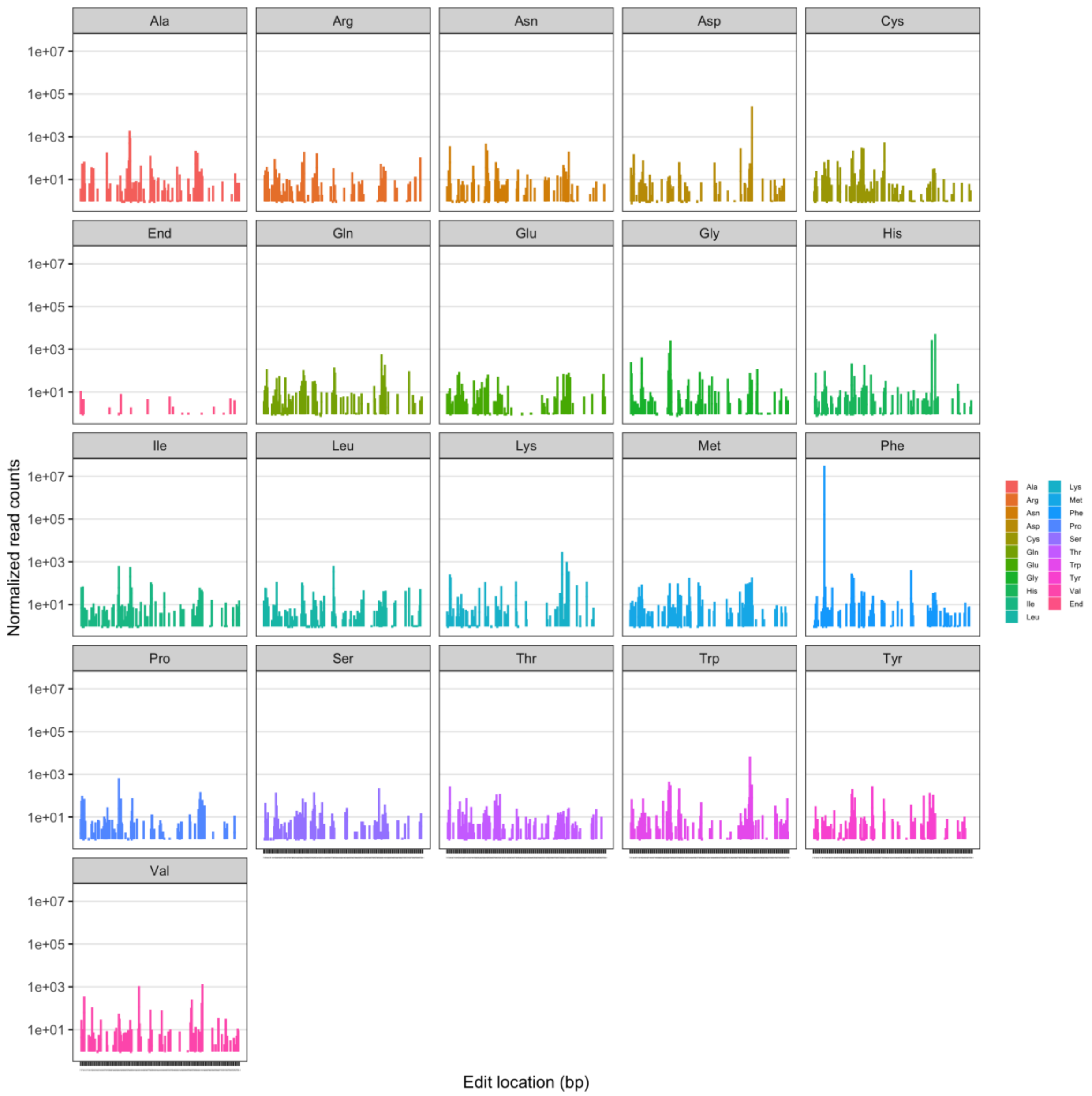


**Figure S11.** Swapped new codons along the full-length endogenous *SPT15* gene partitioned by encoded amino acids, as analyzed from NGS data. The three-letter amino acid abbreviations and the color scheme denote swapped new codons encoding each amino acid.

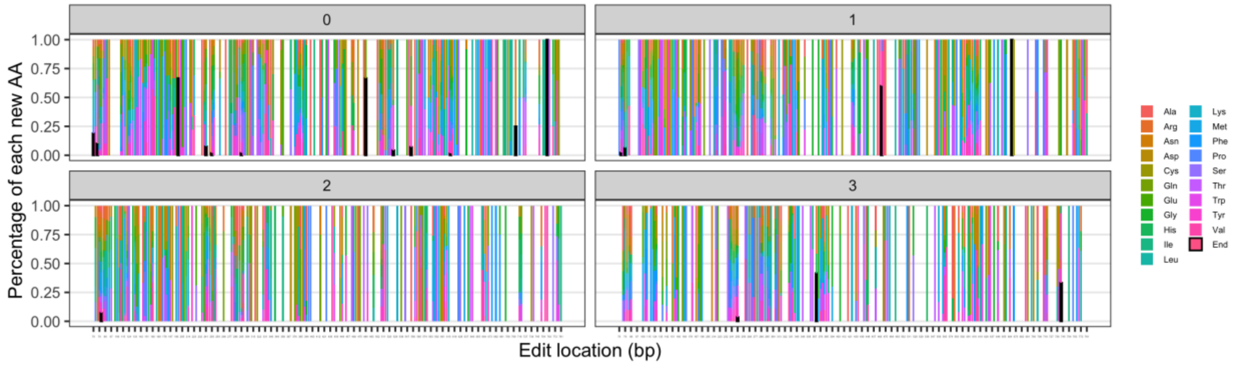


**Figure S12.** Saturation editing of full-length endogenous *SPT15* gene partitioned by the four redundant CHASE cassette designs, as analyzed from NGS data. The numbers 0, 1, 2, and 3 denote CHASE cassettes 0, 1, 2, 3 for each new codon at each codon location. The y axis denotes relative percentage of swapped new codons encoding each new amino acid at each codon location on the x axis. The color scheme denotes swapped new codons encoding each new amino acid. Observed stop codons are denoted with additional bold black borders.

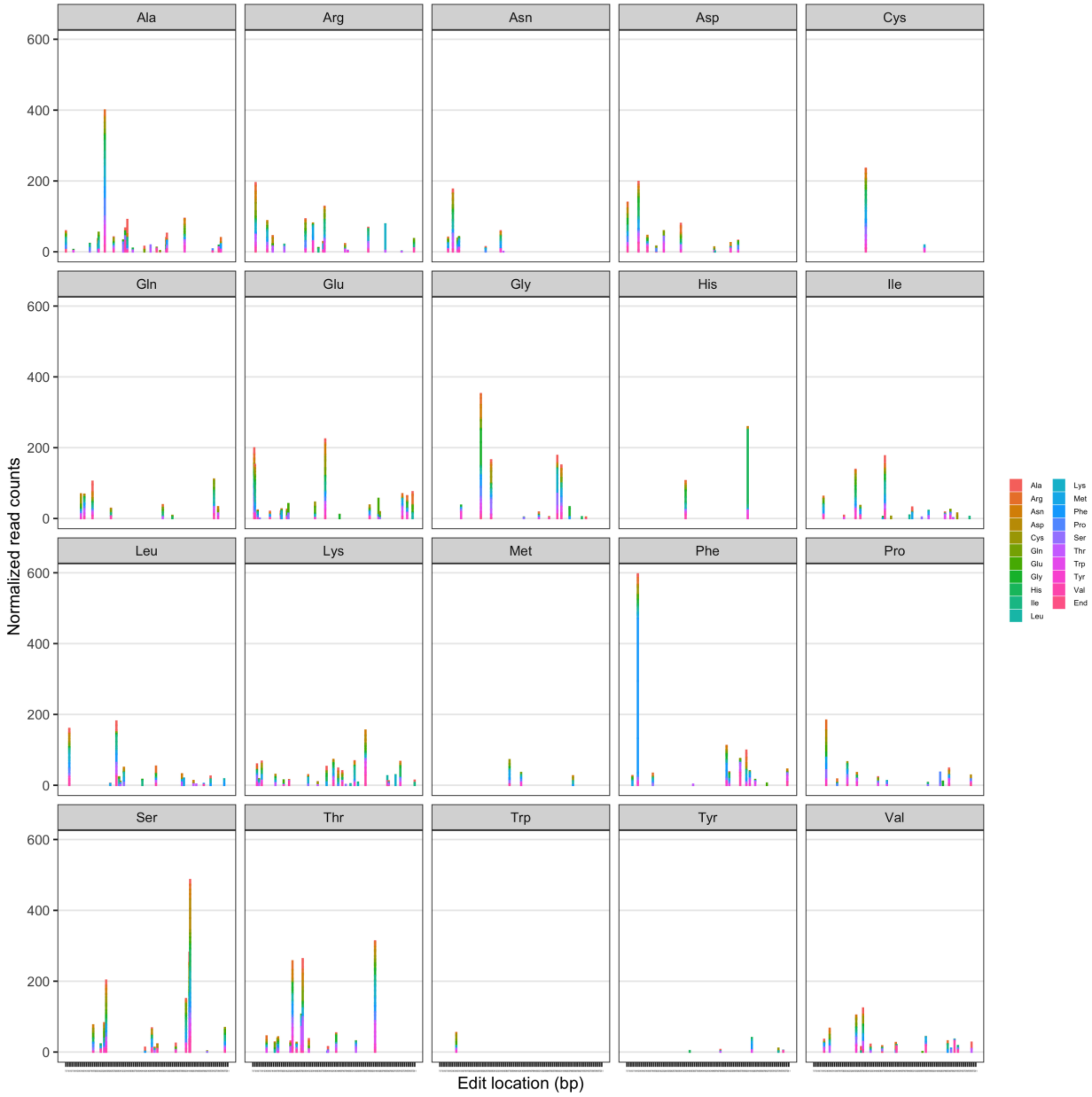


**Figure S13.** Editing of *SPT15* wild type codons by CHASE, as analyzed from NGS data. The data were partitioned into sub panels according to the original wild type amino acids, which were denoted by the three-letter amino acid abbreviations above each sub panel. The color scheme denotes swapped new codons encoding each amino acid.

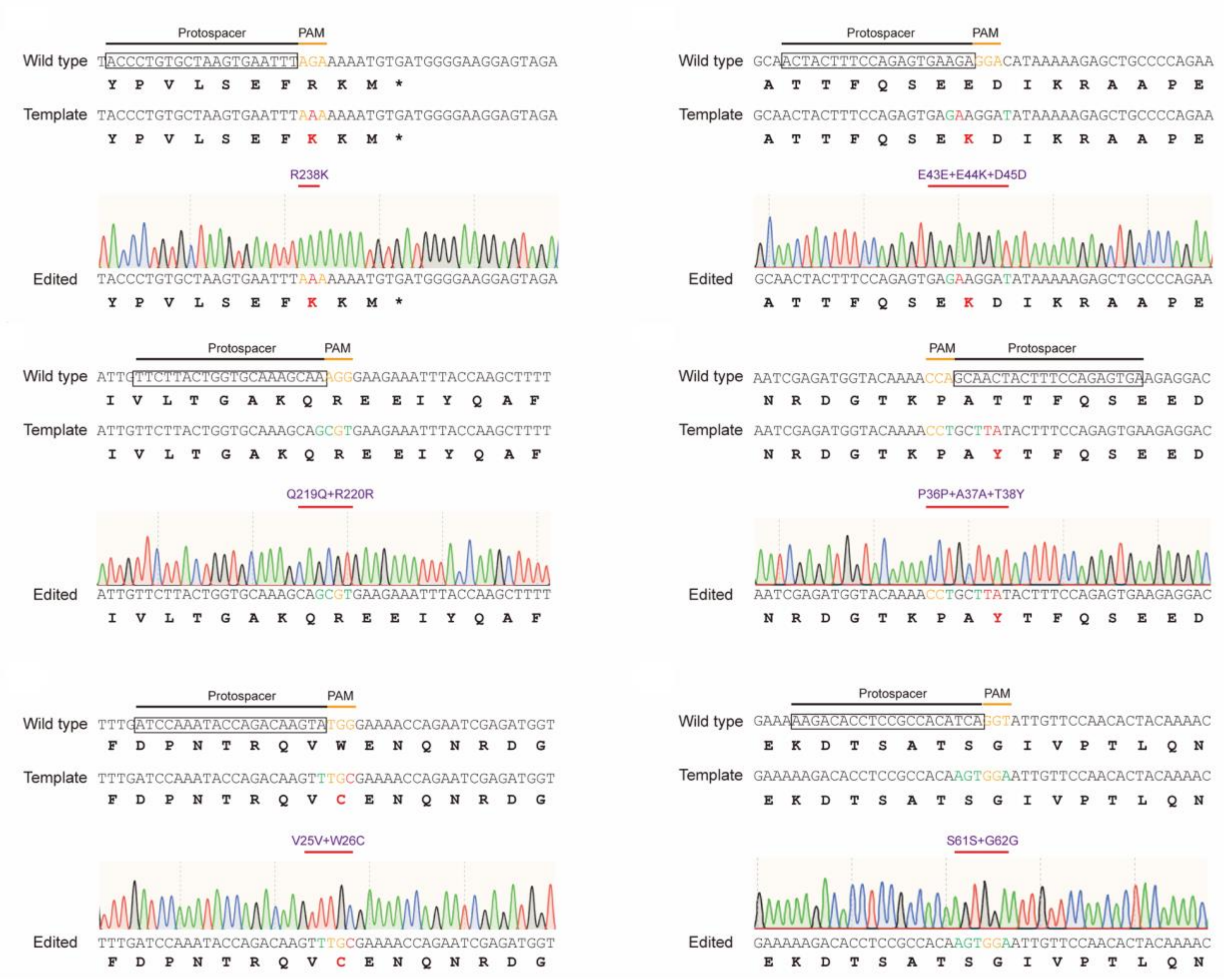


**Figure S14.** Sanger sequencing results of identified *SPT15* mutants from 250 g/L glucose stress screening. The protospacer and PAM sequences, donor template sequence, and Sanger sequencing trace file were shown for each identified colony. Red fonts indicate codon and amino acid mutations. Green letters indicate designed synonymous mutations. Translated protein sequences were shown under each nucleotide sequence.

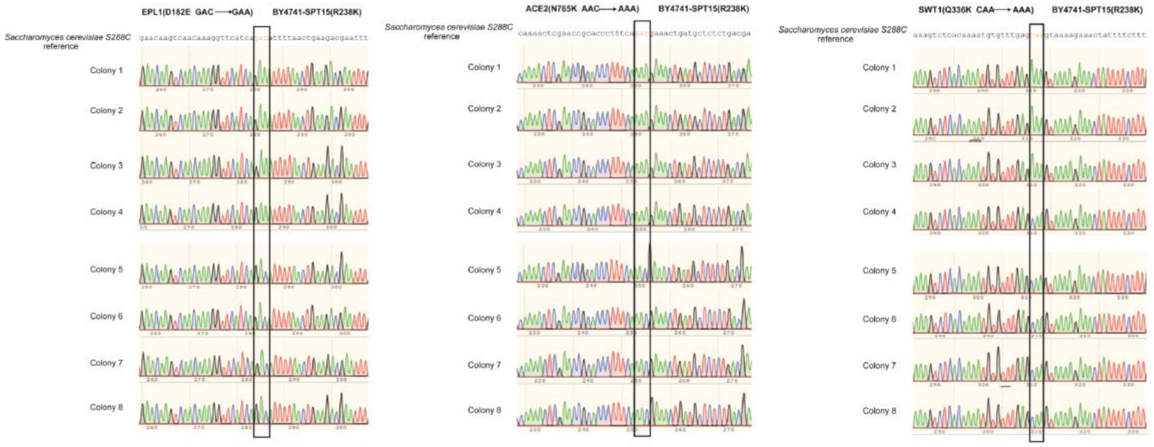


**Figure S15.** Sanger sequencing results of eight wild type BY4741 colonies at the three identified SNP loci of R238K mutants from whole genome sequencing. The SNP loci were denoted by black boxes. For all three loci, both codons were observed in wild type strains, indicating that these loci represent polymorphisms in the background, not de novo mutations generated by off-target activity of SpiG.

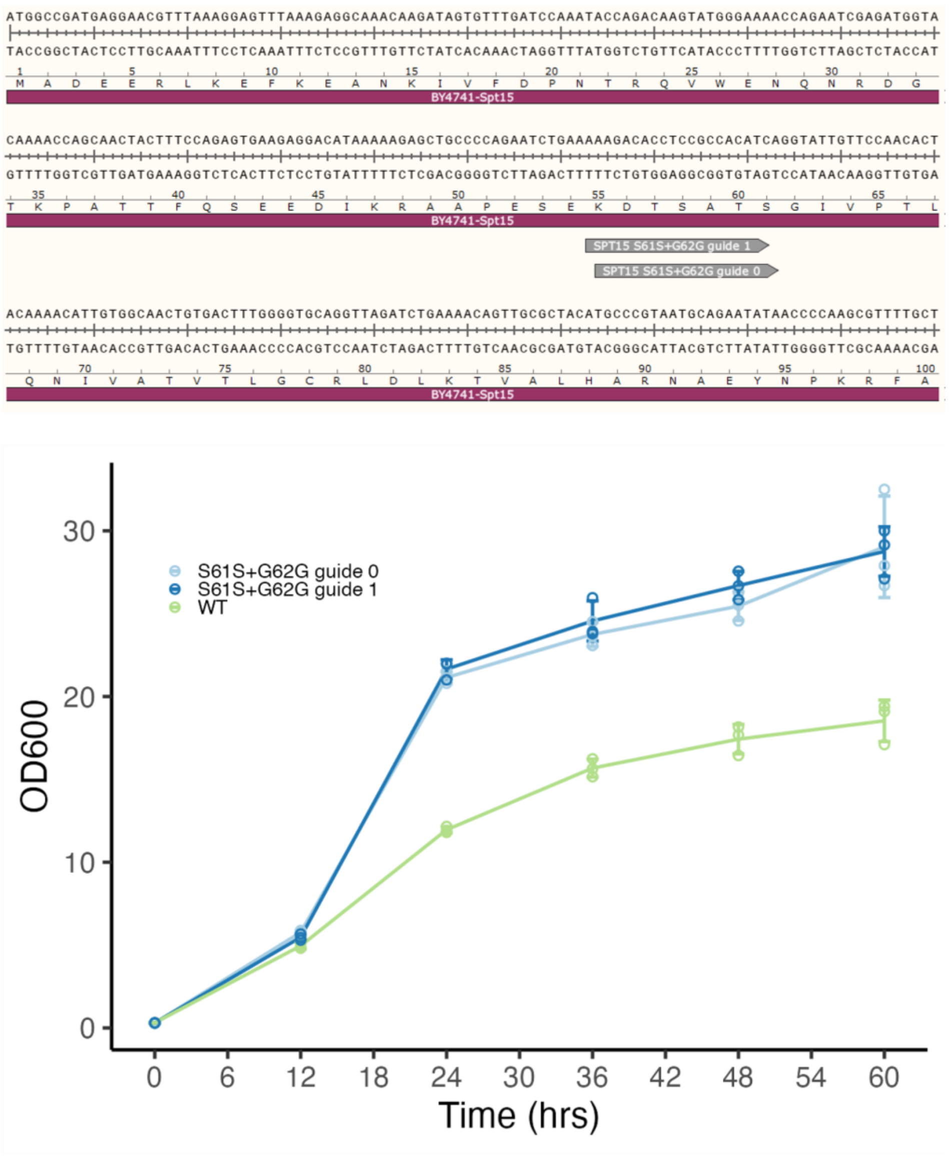


**Figure S16.** Growth profiles of recreated *SPT15* S61S+G62G mutants in YPAD with 250 g/L glucose. The parent WT colony does not have the three second site mutations identified through whole genome sequencing. Guide 0, the original gRNA used in the CHASE library. Guide 1, a second gRNA to make the same S61S+G62G mutation. WT, wild type. n = 3 biological replicates. Error bars represent standard deviations.

## SUPPLEMENTARY TABLES

| Codon # | WT AA | New AA | Cassette # |
|---|---|---|---|
| 12 | Glu | Asn, Lys | 1, 2, 3 |
| 100 | Ala | Cys, Stop, Trp, Tyr | 3 |
| 100 | Ala | Phe | 1, 2, 3 |
| 117 | Ala | Phe | 0, 1, 2, 3 |
| 149 | Ala | Phe | 2, 3 |
| 223 | Ile | All 20 AAs and Stop | 0, 1, 2, 3 |
| 228 | Glu | Phe | 2, 3 |
| 238 | Arg | Phe | 0, 1, 2, 3 |

**Table S5.** Missing cassettes in the *SPT15* CHASE library.

## SEPARATE SUPPLEMENTARY FILES

**Table S1.** A summary of 16 NGN guides.
**Table S2.** A list of 996 NGN CHASE cassettes.
**Table S3.** A list of 618 NGG cassettes.
**Table S4.** A list of 19967 *SPT15* CHASE cassettes.
**Table S6.** Identified non-*SPT15* mutations from whole genome sequencing.
**Table S7.** Primers used in this study.
